# Locomotion difficulty and need for home care: a cross-sectional study

**DOI:** 10.11606/s1518-8787.2025059006939

**Published:** 2025-12-01

**Authors:** Rogério da Silva Linhares, Elaine Thumé, Maria Laura Vidal Carrett, Everton José Fantinel, Maria Aurora Dropa Chrestani Cesar, Elaine Tomasi

**Affiliations:** I Universidade Federal de Pelotas. Faculdade de Medicina. Departamento de Medicina Social. Pelotas, RS, Brasil; II Universidade Federal de Ciências da Saúde de Porto Alegre. Departamento de Saúde Pública. Porto Alegre, RS, Brasil

**Keywords:** House Calls, Mobility Limitation, Equity in Access to Health Services

## Abstract

**OBJECTIVE:**

To investigate home visits by physicians or nurses for consultations and procedures and associated factors in families with people with mobility difficulties and need for home care.

**METHODS:**

A cross-sectional study was carried out with data from *Ciclo III do Programa Nacional de Melhoria do Acesso e da Qualidade da Atenção Básica* (Cycle III of the National Program for Access and Quality Improvement in Primary Care) from September 2017 to June 2018, interviewing over 140,000 users in 28,939 basic health units in 5,312 municipalities. This study analyzed home visits to individuals with mobility difficulties and the need for home care according to internal and external factors via a Poisson regression.

**RESULTS:**

Of all primary healthcare service users, 7.8% stated that someone in their home had mobility difficulties and needed home care. About 70% received home visits. The highest prevalence of home visits, after adjusted analysis, occurred in the Brazilian Northeast. The greater the social vulnerability, the lower the prevalence of home visits in the case of mobility difficulties. Municipalities with 100% coverage from the family health strategy showed a higher prevalence of home visits than those with coverage below 50%.

**CONCLUSION:**

External factors, such as income and social vulnerability index (which have a greater relation with macrosocial determinants), continue to point toward inequalities in care; whereas internal factors, such as family health coverage, community health agent coverage in all micro-areas, and the possibility of receiving care without prior appointments indicated better results. This study reinforces the importance of public policies that encourage complete family health teams to provide comprehensive care to the population.

## INTRODUCTION

In Brazil, the growth of the population coverage under the Family Health Strategy (FHS)^1,2^ and the implementation of the *Programa Melhor em* Casa (Better at Home Program) configure initiatives that highlight the expansion of access to home care in the last decade^3^. Home care belongs to the Health Care Network. It has a set of actions for disease prevention and treatment, rehabilitation, health palliation and promotion, and a guarantee of continuity of care^4^.

Home care contributes to improving individuals’ health, supporting family members and reaffirming the commitment of the Brazilian Unified Health System (SUS) to universal access, comprehensive care, and a focus on the family^5,6^. The home care provided by primary health care (PHC) teams has grown in the last decade after the publication of the national home care policy, which defined the teams’ attributions and the complementarity of network care despite persisting communication problems^7^.

Population-based studies with older adults in Brazil in the last 20 years indicate an increase in palliative care^4,6,8^and the number of people receiving home care. The main reasons for the latter include stroke sequelae, dementia, incapacity for activities of daily living, recent hospitalizations, falls in the previous year, and depression.

A study with data from cycles 1 and 3 of the *Programa de Melhoria do Acesso a Qualidade* (PMAQ – National Program for Improving Access and Quality of Primary Care)—carried out in 2012 and 2017, respectively—shows that more than 98% of the teams reported carrying out home visits in both cycles and an increase in the proportion of teams that carried out such visits, the periodicity of which—defined by a risk and vulnerability analysis —increased from 92.9% (95%CI 92.5–93.3) to 98.4% (95%CI 98.2–98.5). However, according to the teams, they organized 70.4% of the demand for home care in 2012, a proportion that fell to 66.8% in 2017^9^.

Mobility difficulties can compromise individuals’ access to health services^10^. The active work of community health agents (CHA) is crucial to find people with mobility difficulties to assist teams in organizing home activities.

This study aims to investigate home visits for consultations and procedures and the associated factors in families with people with mobility difficulties who require home care.

## METHODS

This cross-sectional study belongs to the external evaluation phase of the FHS teams who participated in Cycle III of PMAQ, held in 2017/2018 in Brazil^11^. The evaluation was carried out under the coordination of Higher Education Institutions led by Universidade Federal de Pelotas, Fundação Oswaldo Cruz, Universidade Federal da Bahia, Universidade Federal de Minas Gerais, Universidade Federal do Piauí, Universidade Federal do Rio Grande do Sul, Universidade Federal do Rio Grande do Norte, and Universidade Federal de Sergipe.

Data were collected from September 2017 to June 2018 by trained interviewers who used an electronic instrument and tablets. Data from interviews with users at basic health units (BHUs) on the day of the evaluation were used for the analyses. The following were chosen as inclusion criteria: age 18 years or older, not visiting the BHU for the first time, having used the service in the 12 months prior to the interview, and reporting someone in their home with mobility difficulties who needed home care. In this cycle, more than 140,000 users linked to 37,350 teams were interviewed in 28,939 BHUs in 5,312 Brazilian municipalities^12^. No size calculation was performed *a priori* for this consecutive sample.

The outcome—home visits for consultations and/or procedures—was constructed from two questions: 1) Is the person with mobility difficulties visited at home by professionals from this BHU/health center (physicians, nurses) for consultations? and 2) Is the person with mobility difficulties visited at home by professionals from this BHU/health center for procedures (dressings, blood collection, vaccines, and others)?

In this study, three contextual characteristics of the municipalities (region [North, Northeast, Southeast, South, and Midwest], the Social Vulnerability Index [SVI; very low, low, medium, high, and very high]^13^, and proportion of FHS population coverage [up to 50%, 50.1% to 75%; 75.1% to 99% and 100%]); teams’ responses about the existence of areas without CHA cover in the territory (yes, no); and two user variables (monthly income [up to 0.50; 0.51 to 1.0; and above 1.0 minimum wage per capita] and the possibility of making an appointment on any day and at any time [yes, no]) were used as exposure variables.

After treating the variables, a descriptive analysis of the sample was conducted with absolute numbers and proportions; then, a bivariate analysis was performed using crude and adjusted prevalence ratios according to a hierarchical model using Poisson regression with robust variance adjustment. The variables for region, SVI and FHS coverage were included at the first more distal level; the variables for area without CHA cover and family income, at the second level; and the possibility of making an appointment at any day and time, at the more proximal third level. Results include proportions, prevalence ratios, and 95% confidence intervals.

The research project was submitted and approved by the Research Ethics Committee of the School of Medicine at Universidade Federal de Pelotas under opinion no. 2.453.320. All interviewees signed an informed consent form stating that they were duly informed about the research topic and guaranteeing secrecy and confidentiality of the provided information and their right to withdrawal from this study at any time.

## RESULTS

A total of 140,299 users participated in the third cycle of the PMAQ external evaluation. This study only included individuals who answered having someone with mobility difficulties and who needed care at home, totaling 10,971 users (7.8%).

The Southeast and Northeast concentrated 70% of the sample, and about two-thirds of participants lived in municipalities with more than 75% FHS coverage (Table 1).

**Table 1:** Description of the users who reported having someone in their home with mobility difficulties and who needed home care according to municipal characteristics, areas without CHA coverage in the territory, and user’s variables. Brazil, 2018 (n = 10,971).

Characteristic	n	%
Region (n = 10,946)		
North	792	7.2
Northeast	3,878	35.4
Midwest	777	7.1
Southeast	3,890	35.5
South	1,609	14.7
Social Vulnerability Index		
Very Low	1,139	10.4
Low	4,049	36.9
Medium	2,633	24.0
High	1,955	17.8
Very high	1,195	10.9
FHS coverage		
Up to 50%	1,840	16.8
> 50% to 75%	2,301	21.0
> 75% to 99%	2,260	20.6
100%	4,570	41.7
Area witout CHA coverage		
Yes	4,405	40.5
No	6,473	59.5
Household income per capita (MW)		
Up to 0,5	5,964	64.3
0,51 to 1	2,687	29.0
Above 1	613	6.6
Possibility of making an appointment at any day and time		
Yes	6,262	61.8
No	3,869	38.2

FHS: family health strategy; CHA: community health agents; MW: minimum wage.

A little more than 40% (n = 4,405) of users received care from teams who had areas without CHA coverage. Investigating household income per capita showed that 64.3% of volunteers (n = 5,964) received up to half a minimum wage and only 6.6% (n = 613), above one minimum wage. Of the total, 61.8% (n = 6,262) of interviewees reported being able to make an appointment every day and at any time during BHU working hours (Table 1).

When asked if they would recommend the BHU to a friend or family member, 90.2% (n = 9,776) answered affirmatively (data not tabulated).

Of all users who answered having a person with mobility difficulties in their homes, 68.2% (n = 7,419) received home visits from BHU professionals for consultations, and 61.7% (n = 6,640) received home visits for procedures. In total, 72.3% (n = 7,851) of users reported visits for consultations or procedures.

After adjusting for the confounding variables and using the North as a reference, the Northeast showed the highest prevalence of home visits, whereas the Midwest, the lowest one. Despite the trend for higher prevalence in the Southeast and South than in the North, differences were insignificant. The greater the social vulnerability of the municipality, the lower the prevalence of home visits in case of mobility difficulties. Municipalities with 100% family health strategy coverage showed a higher prevalence of home visits than those with coverage below 50%. However, municipalities with coverage from 50% to 75% had a lower prevalence of home visits than the reference. Those with coverage from 75% to 99% showed no difference (Table 2).

**Table 2:** Crude and adjusted prevalence ratios for visits in case someone in the residence had mobility difficulties and needed home care. Brazil, 2017–2018.

Variables	n (%)	PR (95%CI)	PR_a_ (95%CI)
Region			
North	516 (66.0)	Reference	Reference
Northeast	2,874 (74.7)	1.13 (1.07–1.19)	1.09 (1.03–1.15)
Midwest	492 (64.0)	0.96 (0.90–1.04)	0.92 (0.85–0.99)
Southeast	2,766 (71.9)	1.09 (1.03–1.15)	1.03 (0.97–1.09)
South	1,192 (75.1)	1.13 (1.07–1.20)	1.03 (0.97–1.11)
Social Vulnerability Index			
Very Low	903 (79.8)	Reference	Reference
Low	2,838 (71.0)	0.89 (0.83–0.96)	0.89 (0.86–0.93)
Medium	1,828 (70.2)	0.88 (0.81–0.95)	0.85 (0.81–0.89)
High	1,391 (71.7)	0.90 (0.83–0.98)	0.81 (0.76–0.85)
Very high	891 (74.9)	0.94 (0.86–1.03)	0.83 (0.79–0.89)
FHS coverage			
Up to 50%	1,299 (71.3)	Reference	Reference
> 50% to 75%	1,515 (66.8)	0.94 (0.90–0.98)	0.92 (0.89–0.96)
> 75% to 99%	1,551 (69.5)	0.97 (0.95–1.01)	0.97 (0.93–1.01)
100%	3,486 (76.8)	1.08 (1.04–1.11)	1.09 (1.05–1.13)
Area witout CHA coverage			
Yes	2,993 (68.7)	Reference	Reference
No	4,814 (75.1)	1.09 (1.07–1.12)	1.05 (1.02–1.08)
Household income per capita (MW)^a^			
Up to 0.5	4,190 (70.9)	Reference	Reference
0.51 to 1	2,031 (76.2)	1.07 (1.04–1.10)	1.09 (1.06–1.12)
Above 1	472 (78.0)	1.10 (1.05–1.15)	1.10 (1.05–1.16)
Possibility of making an appointment at any day and time			
Yes	4,831 (77.8)	Reference	Reference
No	2,433 (63.7)	0.82 (0.80–0.84)	0.83 (0.81–0.85)

FHS: family health strategy; CHA: community health agents; MW: minimum wage; 95%CI: 95% confidence interval; PR: prevalence ratio.

^a^ MW value = R$ 954.00.

PHC teams in areas without CHA coverage made more home visits for consultations and/or procedures than those with uncovered areas (PR = 1.05; 95%CI 1.02–1.08). Families with income per capita above one minimum wage received more home visits for consultations and/or procedures than families earning less than a half minimum wage (PR = 1.10; 95%CI 1.05–1.16) (Table 2).

Users who reported being unable to make an appointment on any day and time had a lower prevalence of home visits for consultations and/or procedures than those who could make an appointment on any day and time (PR = 0.83; 95%CI 0.81–0.85) (Table 2).

## DISCUSSION

This study found that 7.8% of all primary care service users stated that someone in their homes had mobility difficulties and needed home care. It also observed that 72.3% received home visits from physicians or nurses for consultation or procedures; 68.2% received home visits for consultations and 61.7%, for procedures.

Rajão and Martins^14^ analyzed the 4,008,692 home-based procedures performed from November 2012 to December 2016 in the SUS Outpatient Information System. Circulatory diseases accounted for 30.9% of all procedures. Of all home procedures, consultations occurred most often (18.4%), followed by procedures with a multiprofessional team (13.6%), home care by a mid-level professional (12.6%), home visits by a mid-level professional (10.8%), dressings (10.4%), and physical therapy (6.3%).

Nunes et al.^15^, in a nationwide study with CHAs, investigated teams’ criteria for home visits, finding that one third of agents mentioned visiting people with mobility difficulties (53.7%) and chronic diseases (35.9%), older adults (31.1%) and children under two years of age (30.7%) as a priority.

Difficulty moving hinders access to health services. Home care offers an alternative to respond to this need. The literature review in Braga et al.^16^has reported that, despite no measurement of home care demand, the growing offer of these services has failed to meet the various modalities of this quantitative and qualitative demand.

In this study, the most positive findings for the Brazilian Northeast stem from its greater and long-lived FHS coverage. The Community Health Agents Program and the Family Health Program were born and strengthened as a model of care in that region in the early 1990s.

This favorable scenario accentuated the link with PHC principles, especially longitudinal care and extramural activities in the territories (which include home care). A recent study on home visits by CHAs highlights more positive results for the Brazilian Northeast^17^.

The greater the social vulnerability of the municipality, the lower the prevalence of home visits in case of mobility difficulties (with a clearly significant dose-response relationship). This finding may indicate flaws in fundamental principles of SUS (such as equity) as it seems that those who need it most receive such care the least. This study assessed vulnerability via SVI. Its several indicators could make it difficult to interpret its results since the index calculates them for each municipality rather than BHU scope.

Another highlight of this study refers to its higher proportion of home care due to health needs in the municipalities with wider FHS coverage, which can stem from the greater adherence of teams to the attributes of family health, including territorialization and comprehensive longitudinal care^18^. The development of actions in the territory—including home care—is in line with the principle of care integrality, which aims to integrate preventive, curative, and health promotion actions for individuals and communities.

In the FHS model and its characteristic closeness to people’s homes, their families, and community contexts, the territories under the responsibility of its teams configure privileged *loci* for integral and longitudinal care. Municipalities with smaller populations show the widest FHS coverage, which can also contribute to enabling teams to include activities beyond the walls of the health unit in their work processes^19,20^.

Still on the FHS model, receiving home care in case of need occurred more often when the teams reported no areas without CHA coverage. The work of the CHA is organized into micro-areas and characterized by finding the population’s needs and supporting health teams, an important link in the work process^21^. However, many teams still work with a portion of their assigned area without CHAs due to their insufficient numbers or other organizational obstacles. When the teams have no such problems, the work in the territory is more effective, a finding inscribed in this context since it is likely that users linked to teams with areas without CHA coverage reported fewer home visits in cases of family members with mobility difficulties. Areas without CHA coverage may show a population with fewer health promotion and disease prevention activities, imposing greater spontaneous demand on teams for the care of acute events. A recent publication on CHAs’ work quality^22^ found that it is higher in teams in areas with CHA coverage, extending the benefit of complete teams to quality indicators.

Regarding longitudinality, estimates suggest that a large portion of users with mobility difficulties may suffer from chronic non-communicable diseases, such as hypertension, diabetes, obesity, psychological distress, or functional disability, in addition to being older and enduring cardiovascular event sequelae^23^. These and many other conditions increase these residents’ vulnerability. So, home care can facilitate the necessary continued care.

Another PHC characteristic refers to its services configuring a gateway to the health system, that is, the population initially seeks BHUs for care for their health needs. This proportion reached 46.8% of respondents to the 2019 National Health Survey^24^. Preliminary analyses with data from the third cycle of PMAQ indicate that 90% of interviewees referred to their BHU as the service of first choice in case of need (unpublished data). Thus, if it is impossible to go to the BHU due to temporary or permanent limitations, these people will depend on the support of CHAs for their identification and that of medical and nursing professionals for the necessary care and conduct.

The results according to socioeconomic condition—via the income variable per capita —evinced possible equity problems since home visits for procedures and/or consultations occurred more often in families with higher income than in those with lower income (in line the SVI findings for municipalities). However, a study on factors associated with home visits based on PMAQ data found other results: the higher the family income, the lower the number of CHAs performing home visits, which is in line with the principle f equity^17^.

Finally, being able to make an appointment at any day and time was associated with a higher prevalence of receiving home visits when needed. This finding can be attributed to the characteristics of PHC, which sets BHUs as a gateway with guaranteed accessibility for the population. The possibility of scheduling an appointment on any day and at any time signal patients’ possibility of receiving care without prior appointments (which PHC sometimes fails to offer). Management should encourage this practice as it increases the BHU coverage of demand and extends this benefit to home care.

The limitations of this study include its lack of information on the nature of mobility difficulty—whether temporary (lower limb fracture or amputation) or permanent (such as sequelae of stroke, loss of vision, or dementia). Other limitations include the information on limited mobility stemming from someone other than whom receives the care, the possibility of measurement bias related to the information collection method, and its cross-sectional design, which, by measuring outcomes and exposures at the same time, may have brought a potential reverse causality bias to the results, the precise estimate of which is impossible. Note the national coverage of this study based on PHC services and its large sample size. Data included responses from users and team professionals and the contextual characteristics of the municipalities, in which we highlight the SVI, a recent index on social vulnerability.

A conclusion of this study concerns the internal and external factors of the governability of BHUs, its teams, and the health system. External factors, such as municipal SVI (which have a greater relation with macrosocial determinants), continue to point to inequalities in care; whereas internal factors (which characterize health systems, such as FHS municipality coverage, and CHA coverage in all micro-areas), and the possibility of patients receiving care without prior appointments that characterizes the teams’ work process indicated better results. These findings are in line with the essential principles of PHC and SUS, especially equity.

This study reinforces the importance of public policies that encourage the change in the number of FHS professionals toward complete teams that can provide comprehensive care to populations, including for people with mobility difficulties and the need for home care. This study suggests increasing the number of CHAs per team to minimize the number of micro-areas outside their scope to find the population in need of home visits and with difficulty accessing BHUs. The number of nurses and physicians must also fit the size of the population and the territory to enable the management of the agenda and the planning and organization of the team to carry out home visits to the population with mobility difficulties and the need for care at home.

## Data Availability

PMAQ microdata are available from: https://drive.google.com/drive/folders/121Oq9_MqQOGd4jTWEoF1B_Tr6RnZA5cF
