# Supplementary material for: Locomotion difficulty and need for home care: a cross-sectional study
Source: Rev Saude Publica. 2025 Dec 1;59:e35. doi: 10.11606/s1518-8787.2025059006939 (PMC12673639; doi:10.11606/s1518-8787.2025059006939)
Supplement: Translation

# Translation: Dificuldade de locomoção e necessidade de atendimento no domicílio: estudo transversal

**Authors:** Rogério da Silva Linhares, Elaine Thumé, Maria Laura Vidal Carrett, Everton José Fantinel, Maria Aurora Dropa Chrestani Cesar, Elaine Tomasi

**Affiliations:** I Universidade Federal de Pelotas. Faculdade de Medicina. Departamento de Medicina Social. Pelotas, RS, Brasil; II Universidade Federal de Ciências da Saúde de Porto Alegre. Departamento de Saúde Coletiva. Porto Alegre, RS, Brasil

**Keywords:** Visita Domiciliar, Limitação da Mobilidade, Equidade no Acesso

## Abstract

**OBJETIVO:**

Investigar a visita domiciliar para consulta e/ou procedimentos e fatores associados em famílias com pessoas com dificuldade de locomoção e necessidade de atendimento no domicílio por médico ou enfermeiro.

**MÉTODOS:**

Foi realizado um estudo transversal com dados do Ciclo III do Programa Nacional de Melhoria do Acesso e da Qualidade da Atenção Básica, entre setembro de 2017 e junho de 2018, que entrevistou mais de 140.000 usuários em 28.939 unidades básicas de saúde de 5.312 municípios. O estudo analisou o recebimento de visita domiciliar a indivíduos com dificuldade de locomoção e necessidade de atendimento no domicílio de acordo com fatores internos e externos, utilizando regressão de Poisson.

**RESULTADOS:**

Do total de usuários dos serviços de atenção primária, 7,8% afirmaram ter alguém com dificuldade de locomoção e necessidade de atendimento domiciliar. Cerca de 70% receberam visita domiciliar. A maior prevalência de visita domiciliar, após análise ajustada, foi na região Nordeste. Quanto maior a vulnerabilidade social, menor a prevalência de recebimento de visita domiciliar em caso de dificuldade de locomoção. Em municípios com 100% de cobertura de estratégia saúde da família houve maior prevalência de visita domiciliar em relação àqueles com cobertura menor que 50%.

**CONCLUSÃO:**

Fatores externos, como renda e índice de vulnerabilidade social, com maior relação com os macro determinantes sociais, permanecem apontando iniquidades na atenção; enquanto fatores internos, como a cobertura de saúde da família, a cobertura de agente comunitário de saúde em todas as microáreas e “porta aberta” como característica do processo de trabalho das equipes, indicaram melhores resultados. O estudo fortalece a importância de políticas públicas que incentivem a recomposição do número de profissionais das equipes de saúde da família, a fim de terem equipes completas e com capacidade de cuidado integral da população.

## INTRODUÇÃO

No Brasil, a expansão na cobertura populacional da Estratégia Saúde da Família (ESF)^1,2^ e a implementação do Programa Melhor em Casa são iniciativas que destacam a ampliação do acesso ao cuidado domiciliar na última década^3^. A atenção domiciliar integra a Rede de Atenção à Saúde e é caracterizada por um conjunto de ações de prevenção e tratamento de doenças, reabilitação, paliação e promoção à saúde, garantindo continuidade de cuidados^4^.

A atenção no domicílio contribui para melhorar as condições de saúde dos indivíduos, apoiar os familiares e reafirmar o compromisso do Sistema Único de Saúde (SUS) com a universalidade do acesso, a integralidade da atenção e o enfoque familiar^5,6^. O cuidado domiciliar realizado pelas equipes da atenção primária à saúde foi ampliado na última década após a publicação da política nacional de atenção domiciliar, com definição das atribuições das equipes e a complementaridade do cuidado em rede, embora persistam problemas de comunicação^7^.

Estudos de base populacional com idosos no Brasil, nos últimos 20 anos, apontam crescimento no recebimento de atenção domiciliar e os principais motivos foram as sequelas de acidente vascular cerebral, demência, incapacidade para atividades da vida diária, história de hospitalização recente, queda no último ano e depressão, além de cuidados paliativos^4,6,8^.

Estudo com dados dos ciclos 1 e 3 do Programa de Melhoria do Acesso e Qualidade (PMAQ) realizado, respectivamente, em 2012 e 2017, mostra que mais de 98% das equipes informaram realizar visita domiciliar em ambos os ciclos e um aumento na proporção de equipes que realizavam visita domiciliar com periodicidade definida a partir da análise de risco e vulnerabilidade, de 92,9% (IC95% 92,5–93,3) para 98,4% (IC95% 98,2–98,5). Entretanto, ao questionar a equipe sobre a organização da demanda por cuidados domiciliares, a proporção era de 70,4% em 2012 e caiu para 66,8% em 2017^9^.

A dificuldade de locomoção pode comprometer o acesso dos indivíduos aos serviços de saúde^10^. O trabalho ativo dos agentes comunitários de saúde (ACS) é crucial na identificação de pessoas com dificuldade de mobilidade para auxiliar as equipes na organização das atividades no domicílio.

O objetivo deste artigo foi investigar o recebimento de visita domiciliar para consulta e/ou procedimentos e os fatores associados em famílias com pessoas com dificuldade de locomoção e que consideram necessitar de atendimento no domicílio.

## MÉTODOS

O estudo tem delineamento transversal e é um recorte da fase de avaliação externa de equipes da ESF, que participaram do Ciclo III do PMAQ, realizado em 2017/2018 no Brasil^11^. A avaliação foi executada sob a coordenação de Instituições de Ensino Superior e lideradas por: Universidade Federal de Pelotas, Fundação Oswaldo Cruz, Universidade Federal da Bahia, Universidade Federal de Minas Gerais, Universidade Federal do Piauí, Universidade Federal do Rio Grande do Sul, Universidade Federal do Rio Grande do Norte e Universidade Federal de Sergipe.

A coleta de dados foi realizada no período de setembro de 2017 a junho de 2018 por entrevistadores treinados, utilizando instrumento eletrônico por meio de *tablets*. Para as análises, utilizou-se dados da entrevista com usuários presentes na unidade básica de saúde (UBS) no dia da avaliação. Os critérios de inclusão para os usuários foram ter 18 anos ou mais, não estar sendo atendido na UBS pela primeira vez, ter utilizado o serviço nos 12 meses anteriores à entrevista e referir ter alguma pessoa em sua residência com dificuldade de locomoção e que necessita de atendimento no domicílio. Nesse ciclo foram entrevistados mais de 140 mil usuários vinculados a 37.350 equipes, em 28.939 UBS de 5.312 municípios brasileiros^12^. Trata-se de uma amostra consecutiva, não tendo sido realizado cálculo de tamanho amostral *a priori*.

O desfecho – recebimento de visita domiciliar para consultas e/ou procedimentos – foi construído a partir de duas perguntas: 1) Esta pessoa com dificuldade de locomoção recebe visita de profissionais desta UBS/posto de saúde para realização de consultas (médico, enfermeiro) em sua casa?; e 2) Esta pessoa recebe a visita de profissionais desta UBS/posto de saúde para realização de procedimentos (curativos, coleta de sangue, vacinas e outros) em sua casa?

Utilizou-se como variáveis de exposição três características contextuais dos municípios: região (Norte, Nordeste, Sudeste, Sul e Centro-Oeste), Índice de Vulnerabilidade Social – IVS (muito baixo, baixo, médio, alto e muito alto)^13^ e proporção de cobertura populacional com ESF (até 50%, 50,1 a 75%; 75,1 a 99% e 100%); resposta da equipe sobre a existência de área descoberta por ACS no território (sim, não); e duas variáveis do usuário (renda mensal *per capita* em salários mínimos (até 0,50; 0,51 a 1,0; e acima de 1,0), e se consegue marcar consulta em qualquer dia e qualquer horário (sim, não).

Após a limpeza e edição das variáveis, uma análise descritiva da amostra foi conduzida com números absolutos e proporções; em seguida, procedeu-se à análise bivariada por meio das razões de prevalência brutas e ajustadas segundo modelo hierárquico, utilizando regressão de Poisson com ajuste robusto de variância. No primeiro nível – mais distal – entraram as variáveis região, IVS e cobertura de ESF; no segundo nível, as variáveis área descoberta de ACS e renda familiar; no terceiro nível – mais proximal – poder marcar consulta a qualquer dia e horário. Os resultados incluem proporções, razões de prevalência e intervalos de confiança de 95%.

O estudo foi submetido e aprovado pelo Comitê de Ética em Pesquisa da Faculdade de Medicina da Universidade Federal de Pelotas, que emitiu parecer favorável nº 2.453.320. Todos os entrevistados assinaram um termo de consentimento livre e esclarecido, afirmando que foram devidamente informados sobre o tema da pesquisa, sobre a garantia do sigilo e da confidencialidade das informações prestadas e sobre o direito de recusar a participar a qualquer momento do estudo.

## RESULTADOS

Participaram do terceiro ciclo de avaliação externa do PMAQ 140.299 usuários e, para o presente estudo, foram incluídos apenas os indivíduos que responderam ter em sua residência alguém com dificuldade de locomoção e que necessitava de atendimento no domicílio, totalizando 10.971 usuários (7,8%).

As regiões Sudeste e Nordeste concentraram 70% da amostra e cerca de dois terços residiam em municípios com mais de 75% de cobertura de ESF (Tabela 1).

**Tabela 1:** Descrição dos usuários que responderam ter em sua residência alguém com dificuldade de locomoção e que necessita de atendimento no domicílio conforme características dos municípios, existência de área descoberta por ACS no território e variáveis do usuário. Brasil, 2018 (n = 10.971).

Variáveis	n	%
Região (n = 10.946)		
Norte	792	7,2
Nordeste	3.878	35,4
Centro-Oeste	777	7,1
Sudeste	3.890	35,5
Sul	1.609	14,7
Índice de vulnerabilidade social		
Muito baixo	1.139	10,4
Baixo	4.049	36,9
Médio	2.633	24,0
Alto	1.955	17,8
Muito alto	1.195	10,9
Cobertura de SF		
Até 50%	1.840	16,8
> 50% a 75%	2.301	21,0
> 75% a 99%	2.260	20,6
100%	4.570	41,7
Equipe tem área descoberta de ACS		
Sim	4.405	40,5
Não	6.473	59,5
Renda familiar *per capita* (SM)		
Até 0,5	5.964	64,3
0,51 a 1	2.687	29,0
Acima de 1	613	6,6
Pode marcar consulta a qualquer dia e horário		
Sim	6.262	61,8
Não	3.869	38,2

SF: saúde da família; ACS: agentes comunitários de saúde; SM: salário mínimo.

Pouco mais de 40% (n = 4.405) dos usuários pertenciam a equipes que tinham área descoberta por ACS. Ao investigar a renda familiar *per capita*, 64,3% (n = 5.964) contavam com até meio salário mínimo e apenas 6,6% (n = 613) contavam com valores acima de um salário mínimo. Do total, 61,8% (n = 6.262) dos entrevistados referiram poder marcar consulta todos os dias e em qualquer momento do funcionamento da UBS (Tabela 1).

Ao serem questionados se recomendariam a UBS para amigo ou familiar, 90,2% (n = 9.776) respondeu que recomendaria (dados não tabulados).

Do total dos usuários que responderam ter em seu domicílio alguma pessoa com dificuldade de locomoção, 68,2% (n = 7.419) receberam visita domiciliar de profissionais da UBS para realização de consultas, enquanto 61,7% (n = 6.640) receberam visita para realização de procedimentos. A proporção de usuários que afirmou ter recebido visita para algum tipo de atendimento ou para ambos foi de 72,3% (n = 7.851).

Após ajuste para variáveis de confusão, tendo a região Norte como referência, a maior prevalência de recebimento de visita domiciliar foi na região Nordeste, enquanto a região Centro-Oeste apresentou menor prevalência. Nas regiões Sudeste e Sul, apesar da tendência de maior prevalência que a região Norte, as diferenças não foram significativas. Quanto maior a vulnerabilidade social do município, menor a prevalência de recebimento de visita domiciliar em caso de dificuldade de locomoção. Nos municípios com cobertura de saúde da família de 100% houve maior prevalência de visita domiciliar, em comparação com aqueles com cobertura menor que 50%. No entanto, municípios com cobertura entre 50% e 75% apresentaram menor prevalência de visita domiciliar em relação à referência, e aqueles com cobertura entre 75% e 99% não apresentaram diferença (Tabela 2).

**Tabela 2:** Razões de prevalência brutas e ajustadas para recebimento de algum tipo de visita em caso de ter na residência alguém com dificuldade de locomoção e necessidade de atendimento domiciliar. Brasil, 2017–2018.

Variáveis	n (%)	RP (IC95%)	RP_a_ (IC95%)
Região			
Norte	516 (66,0)	Referência	Referência
Nordeste	2.874 (74,7)	1,13 (1,07–1,19)	1,09 (1,03–1,15)
Centro-Oeste	492 (64,0)	0,96 (0,90–1,04)	0,92 (0,85–0,99)
Sudeste	2.766 (71,9)	1,09 (1,03–1,15)	1,03 (0,97–1,09)
Sul	1.192 (75,1)	1,13 (1,07–1,20)	1,03 (0,97–1,11)
Índice de vulnerabilidade social			
Muito baixo	903 (79,8)	Referência	Referência
Baixo	2.838 (71,0)	0,89 (0,83–0,96)	0,89 (0,86–0,93)
Médio	1.828 (70,2)	0,88 (0,81–0,95)	0,85 (0,81–0,89)
Alto	1.391 (71,7)	0,90 (0,83–0,98)	0,81 (0,76–0,85)
Muito alto	891 (74,9)	0,94 (0,86–1,03)	0,83 (0,79–0,89)
Cobertura de SF			
Até 50%	1.299 (71,3)	Referência	Referência
> 50% a 75%	1.515 (66,8)	0,94 (0,90–0,98)	0,92 (0,89–0,96)
> 75% a 99%	1.551 (69,5)	0,97 (0,95–1,01)	0,97 (0,93–1,01)
100%	3.486 (76,8)	1,08 (1,04–1,11)	1,09 (1,05–1,13)
Equipe tem área descoberta de ACS			
Sim	2.993 (68,7)	Referência	Referência
Não	4.814 (75,1)	1,09 (1,07–1,12)	1,05 (1,02–1,08)
Renda familiar *per capita* (SM)^a^			
Até 0,5	4.190 (70,9)	Referência	Referência
0,51 a 1	2.031 (76,2)	1,07 (1,04–1,10)	1,09 (1,06–1,12)
Acima de 1	472 (78,0)	1,10 (1,05–1,15)	1,10 (1,05–1,16)
Pode marcar consulta a qualquer dia e horário			
Sim	4.831 (77,8)	Referência	Referência
Não	2.433 (63,7)	0,82 (0,80–0,84)	0,83 (0,81–0,85)

SF: saúde da família; ACS: agentes comunitários de saúde; SM: salário mínimo; IC95%: intervalo de confiança de 95%; RP: razão de prevalência.

^a^ Valor do SM = R$ 954,00.

Equipes de atenção primária à saúde (APS) sem área descoberta de ACS realizavam mais visitas domiciliares para consulta e/ou procedimento em comparação com aquelas com área descoberta (RP = 1,05; IC95% 1,02–1,08). Famílias com renda *per capita* maior que 1,0 salário mínimo recebem mais visita domiciliar para consultas e/ou procedimentos do que famílias que recebem menos de 0,5 salário mínimo (RP = 1,10; IC95% 1,05–1,16) (Tabela 2).

Usuários que referiram não poder marcar consulta a qualquer dia e horário apresentaram menor prevalência de visita domiciliar para consulta e/ou procedimento em relação àqueles que podiam marcar consulta a qualquer dia e horário (RP = 0,83; IC95% 0,81–0,85) (Tabela 2).

## DISCUSSÃO

Neste estudo, foi identificado que do total de usuários dos serviços de atenção primária, 7,8% referiram ter no domicílio alguém com dificuldade de locomoção e necessidade de atendimento domiciliar. Foi observado que 72,3% receberam visita domiciliar para consulta ou procedimento por médico ou enfermeiro, sendo que 68,2% receberam visita domiciliar para realização de consultas, enquanto 61,7% receberam visita domiciliar para realização de procedimentos.

Rajão e Martins^14^ realizaram análises dos 4.008.692 procedimentos de âmbito domiciliar realizados no período de novembro de 2012 a dezembro de 2016 registrados no Sistema de Informações Ambulatoriais do SUS (SIA-SUS). As doenças do aparelho circulatório representaram 30,9% dos procedimentos. Do volume total de procedimentos domiciliares, os mais frequentes foram a consulta/atendimento (18,4%), seguido pelos procedimentos por equipe multiprofissional (13,6%), assistência domiciliar por profissional de nível médio (12,6%), visita domiciliar por profissional de nível médio (10,8%), curativos (10,4%) e atendimento fisioterapêutico (6,3%).

Nunes et al.^15^, em estudo de abrangência nacional com ACS, investigaram a existência de critérios para realização de visitas domiciliares definidos pela equipe, e um terço dos agentes mencionaram como prioridade de visita a pessoas com dificuldade de locomoção (53,7%), portadores de doenças crônicas (35,9%), idosos (31,1%) e crianças menores de 2 anos (30,7%).

A dificuldade de locomoção dificulta o acesso aos serviços de saúde e, frente à necessidade de atenção, o atendimento domiciliar é alternativa para responder a essa necessidade. Braga et al.^16^, ao realizar revisão da literatura, referem que, apesar da demanda por atenção domiciliar não estar mensurada, a expansão da oferta desses serviços não tem acompanhado a demanda, tanto quantitativa quanto qualitativa, nas suas diversas modalidades.

Neste estudo, os achados mais positivos para o Nordeste podem ser creditados à maior e mais longeva cobertura da ESF registrada nessa região. Foi lá que, no início da década de 1990, o Programa de Agentes Comunitários de Saúde e o Programa de Saúde da Família nasceram e se fortaleceram como modelo de atenção.

Com esse cenário favorável, acentua-se o vínculo com os princípios da APS, com ênfase na longitudinalidade do cuidado e em atividades extramuro, nos territórios, entre as quais se localiza o cuidado no domicílio. Recente estudo sobre a realização de visitas domiciliares por ACS destacam resultados mais positivos para a região Nordeste^17^.

O estudo verificou que quanto maior a vulnerabilidade social do município, menor a prevalência de recebimento de visita domiciliar em caso de dificuldade de locomoção, em uma clara relação significativa de dose-resposta. Esse achado pode indicar falhas em princípios fundamentais do SUS como a equidade, pois parece que quem mais precisa, menos recebe esse atendimento. A vulnerabilidade foi avaliada pelo IVS, um índice composto por diversos indicadores, o que poderia dificultar a interpretação desses resultados, uma vez que é calculado para o município e não para o âmbito da UBS.

Outro destaque do estudo foi a maior proporção de atendimento domiciliar em caso de necessidade nos municípios com maior cobertura populacional de ESF, o que pode ser atribuído à maior adesão das equipes aos atributos da saúde da família, dentre eles a territorialização, a longitudinalidade e a integralidade do cuidado^18^. O desenvolvimento de ações no território – entre elas o cuidado nos domicílios – vem ao encontro do princípio da integralidade da atenção, que supõe a integração entre ações preventivas, curativas e de promoção de saúde para indivíduos e coletividade.

No modelo da ESF, com sua característica de estar próxima de onde as pessoas vivem, em seus contextos familiares e comunitários, os territórios sob responsabilidade das equipes configuram-se como *locus* privilegiados para a integralidade e a longitudinalidade do cuidado. Sabe-se que as maiores coberturas de ESF estão presentes em municípios de menor porte populacional, o que também pode contribuir para que as equipes consigam inserir nos seus processos de trabalho atividades para além dos muros da unidade de saúde^19,20^.

Ainda relacionado ao modelo da ESF, encontrou-se que o recebimento de cuidado domiciliar em caso de necessidade foi mais frequente quando a equipe referiu não ter áreas descobertas por ACS. O trabalho dos ACS é organizado em microáreas e é caracterizado pela identificação de necessidades da população e pelo apoio às equipes de saúde, sendo um elo importante no processo de trabalho^21^. Entretanto, sabe-se que muitas equipes ainda trabalham com parte da sua área adstrita sem a presença de ACS, por número insuficiente desses profissionais ou outros entraves organizacionais. Quando a equipe não tem esses problemas, o trabalho no território é mais efetivo, e nesse âmbito se inscreve esse achado, pois é provável que usuários vinculados a equipes com áreas descobertas de ACS tenham referido menos recebimento de cuidado domiciliar em caso de algum familiar com dificuldade de locomoção. Áreas descobertas de ACS podem apresentar uma população com menos atividades de promoção de saúde e prevenção de doenças, impondo maior demanda espontânea às equipes para o atendimento de eventos agudos. Recente publicação sobre a qualidade do trabalho dos ACS^22^ encontrou que essa qualidade é maior em equipes que não referem área sem ACS em seu território de abrangência, estendendo o benefício de equipes completas para indicadores de qualidade.

Em relação à longitudinalidade, estima-se que boa parte dos usuários com dificuldade de locomoção possam sofrer de doenças crônicas não transmissíveis, como hipertensão, diabete, obesidade, sofrimento psíquico ou incapacidade funcional, além de serem mais idosos e pessoas com sequela de eventos cardiovasculares^23^. Essas condições – e tantas outras – aumentam a vulnerabilidade desses moradores, de modo que os atendimentos no domicílio podem facilitar o necessário cuidado continuado.

Outra característica da APS é que seus serviços são porta de entrada para o sistema de saúde, ou seja, a população procura inicialmente as UBS em busca de atendimento para suas necessidades de saúde. Essa proporção atingiu 46,8% dos entrevistados pela Pesquisa Nacional de Saúde de 2019^24^. Análises preliminares, com dados do terceiro ciclo do PMAQ, informam que 90% dos usuários entrevistados referiram a sua UBS como serviço de primeira escolha em caso de necessidade (dados não publicados). Assim, na impossibilidade de dirigir-se à UBS, seja por limitação temporária ou permanente, pessoas com esse perfil precisam contar com o apoio dos ACS – para sua identificação – e dos profissionais de medicina e enfermagem para os atendimentos e condutas necessárias.

Os resultados segundo a condição socioeconômica – por meio da variável renda *per capita* – foram na mesma direção dos achados do IVS para o nível dos municípios, remetendo a possíveis problemas de equidade, pois o recebimento de visita domiciliar para procedimentos e/ou consultas foi maior em famílias com maior renda do que entre aquelas com menor renda. Entretanto, estudo sobre fatores associados ao recebimento de visita domiciliar, também com dados do PMAQ, encontrou resultados diferentes: quanto mais alta a renda da família, menor o recebimento de visita domiciliar pelos ACS, o que vai ao encontro do princípio da equidade^17^.

Por fim, o fato de poder marcar consulta a qualquer dia e horário esteve associado a maior prevalência de recebimento de visita domiciliar em caso de necessidade. Esse achado pode ser atribuído às próprias características da APS, que tem na UBS sua porta de entrada com garantia de acessibilidade para a população. A possibilidade de marcação de atendimento em qualquer dia e qualquer horário é sinal de porta aberta, o que nem sempre ocorre na APS. Essa prática deve ser incentivada pela gestão, pois além de ampliar a cobertura sobre a demanda no ambiente da UBS, estende esse benefício ao cuidado domiciliar.

Entre as limitações, podemos citar que não havia informação sobre motivo de dificuldade de locomoção, seja devido a um problema de saúde temporário – fratura ou amputação de membro inferior – ou permanente, como uma sequela de acidente vascular cerebral, perda de visão ou demência. Outra limitação foi que a informação não foi fornecida diretamente pela pessoa com dificuldade de locomoção. Além disso, há possibilidade de viés de aferição relacionado à forma de coleta das informações. Também o delineamento transversal, por aferir desfechos e exposições em um mesmo momento, pode ter trazido um potencial viés de causalidade reversa aos resultados, o que não é possível de estimar com precisão. Em favor do estudo, destaca-se que teve abrangência nacional, com base em serviços de APS, com grande tamanho de amostra. Os dados incluíram respostas dos usuários e dos profissionais das equipes, além de características contextuais dos municípios, com destaque para o IVS, índice recente sobre vulnerabilidade social.

Uma das conclusões deste estudo diz respeito aos fatores internos e externos à governabilidade da UBS, suas equipes e o sistema de saúde. Fatores externos como renda e IVS do município, com maior relação com os macro determinantes sociais, permanecem apontando iniquidades na atenção; já fatores internos, o que é próprio do sistema de saúde, como a cobertura de ESF no município, a cobertura de ACS em todas as microáreas e “porta aberta” como característica do processo de trabalho das equipes, indicaram melhores resultados. Tais achados vão ao encontro dos princípios essenciais da APS e do SUS, com destaque para a equidade.

Este estudo fortalece a importância de políticas públicas que incentivem a recomposição do número de profissionais das equipes de ESF, a fim de terem equipes completas e com capacidade de cuidado integral da população, incluindo as pessoas com dificuldade de locomoção e necessidade de atendimento no domicílio. Em especial, sugere-se o aumento do número de ACS por equipe a fim de minimizar a quantidade de microáreas descobertas de ACS, para poderem identificar a população com necessidade de visita domiciliar e dificuldade de acessar a UBS. Também é necessário que o número de enfermeiros e médicos esteja adequado ao tamanho populacional e do território, para permitir o gerenciamento da agenda e o planejamento e organização da equipe, para realizar visita domiciliar à população com dificuldade de locomoção e que necessitam de atendimento no domicílio.

## Data Availability

Os microdados do PMAQ estão disponíveis no endereço: https://drive.google.com/drive/folders/121Oq9_MqQOGd4jTWEoF1B_Tr6RnZA5cF.
